# Delphinidin Exerts Immunomodulatory Effects in Canine Neutrophils and Peripheral Blood Mononuclear Cells by Limiting Tissue Damaging Mechanisms and Regulating Cytokine Responses

**DOI:** 10.3390/ani16050746

**Published:** 2026-02-27

**Authors:** Alejandra I. Hidalgo, Macarena Vega, Denisse Maldonado, Stefanie Teuber, Rafael A. Burgos, María A. Hidalgo

**Affiliations:** Laboratory of Immunometabolism, Institute of Pharmacology and Morphophysiology, Universidad Austral de Chile, Independencia 631, Valdivia 5690000, Chile; macarena.vega02@alumnos.uach.cl (M.V.); denisse.maldonado@alumnos.uach.cl (D.M.); stefanie.teuber@uach.cl (S.T.); rburgos1@uach.cl (R.A.B.)

**Keywords:** delphinidin, canine neutrophils, mononuclear cells, immunomodulation

## Abstract

Dogs can develop chronic inflammatory diseases that induce progressive tissue damage and illness. Delphinidin is a component of maqui (*Aristotelia chilensis*) and has anti-inflammatory and antioxidant effects. This study evaluated the immunomodulatory effects of delphinidin chloride (DC) and delphinidin-3-glucoside (D3G) on neutrophils and peripheral blood mononuclear cells (PBMCs) in dogs. Leukocytes were isolated from 20 clinically healthy dogs and treated with DC and D3G at concentrations of 50, 100, and 150 µM. The cells were then stimulated with lipopolysaccharide (LPS), platelet-activating factor (PAF), or phorbol 12-myristate 13-acetate (PMA) to evaluate cell viability, reactive oxygen species (ROS) production, neutrophil extracellular trap (NET) formation, phagocytosis, chemotaxis, matrix metalloproteinase 9 (MMP-9) activity, and cytokine production. The results showed that both compounds preserved cell viability, significantly reducing ROS production and NET formation. DC significantly increased chemotaxis and D3G significantly reduced MMP-9 activity. Both compounds reduced the secretion of interleukin (IL) 1β (IL-1β) and tumor necrosis factor α (TNF-α) in neutrophils. In PBMCs, they decreased the production of IL-4 and IL-6 and modulated the production of interferon γ (IFN)-γ. In conclusion, delphinidin exerts selective anti-inflammatory activities in canine leukocytes, promoting inflammation resolution, suggesting its potential role as a nutraceutical for managing inflammatory pathologies in dogs.

## Simple Summary

Diseases that cause chronic inflammation severely affect the quality of life of dogs by damaging their tissues and causing premature aging. Currently, natural alternatives are being sought to treat these problems without the side effects of traditional medications. In this context, delphinidin, a bioactive compound present in maqui berries, is emerging as a promising candidate for treating these conditions in dogs, although there is still limited scientific evidence regarding its efficacy. This study aimed to evaluate whether delphinidin regulates the canine immune system. When tested on canine blood cells, the results showed that this compound does not affect cell viability, increases cell migration, and acts by reducing mechanisms that cause tissue damage, as it decreases the production of reactive oxygen species, formation of extracellular traps, and activity of the enzyme metalloproteinase 9. Simultaneously, it regulated the production of inflammatory cytokines, decreasing the production of interleukin 1β, tumor necrosis factor α, and interleukin 6, and increasing the production of interferon-γ. In conclusion, delphinidin effectively balanced the immune response in dogs. These findings support the study and development of new naturally occurring compounds that can be used as supplements to prevent and treat inflammatory diseases in dogs.

## 1. Introduction

The immune system is fundamental for defending organisms against injuries and microorganisms and maintaining physiological homeostasis [1]. Its main components include leukocytes and specialized cells that reside in and explore tissues. These cells are divided into granulocytes or polymorphonuclear cells (PMN), which include neutrophils, eosinophils, and basophils [2], and peripheral blood mononuclear cells (PBMCs), a group formed by lymphocytes, monocytes, natural killer (NK) cells, and dendritic cells [3].

The canine immune system is complex, with differential susceptibility among breeds to infectious diseases and autoimmune disorders [4]. The activation of this system usually responds to infections and dermal conditions [5,6,7], where the balance between the protective response and tolerance is critical [8]. Therefore, understanding its regulation is key to developing new vaccines and therapies [9,10].

Neutrophils, the most abundant leukocytes [11], act as the first line of defense [12] through mechanisms that, although protective, can cause tissue damage if dysregulated [13]. Among these mechanisms, the production of reactive oxygen species (ROS), such as superoxide anions, stands out as it can damage extracellular targets and neighboring cells [14]. Likewise, they release granular enzymes such as matrix metalloproteinase 9 (MMP-9), an endopeptidase that facilitates leukocyte recruitment and extracellular matrix remodeling, but which, in chronic phases, contributes to disease persistence [15,16]. Additionally, neutrophils release extracellular traps (NETs), structures of chromatin and proteins that trap pathogens but can also aggravate inflammation and tissue damage [17,18].

Although classically associated with innate immunity, neutrophils orchestrate the adaptive response by communicating with dendritic cells and PBMCs [19,20], mainly through cytokine secretion. Cytokines are essential for cell activation and migration [21] and are therapeutic targets in several diseases, such as cancer and autoimmune diseases [22]. Inflammatory cytokines, such as interleukin (IL)-1β, IL-6, and tumor necrosis factor (TNF)-α, play central roles in the acute phase, fever, and tissue repair; however, their excess is associated with severe pathologies in dogs, such as acute pancreatitis [23], sepsis [24], and autoimmune diseases, such as pemphigus foliaceus [25]. Similarly, the chemokine IL-8 is a potent neutrophil attractant implicated in inflammatory processes and tumor neovascularization [26,27]. On the other hand, cytokines such as IL-17A, IFN-γ, and IL-4 regulate specific responses against pathogens and allergic processes like atopic dermatitis, although their exact roles in canines can be contradictory or dependent on the pathological context [28,29].

Given the need for therapeutic alternatives that balance canine immunity without the adverse effects of traditional drugs, the use of natural compounds has gained relevance [30]. This fact is because non-steroidal anti-inflammatory drugs (NSAIDs) in dogs can cause gastrointestinal ulceration, liver damage [31,32] and acute renal failure [33]. Furthermore, glucocorticoid administration in dogs can cause adverse effects such as polyuria, polydipsia, polyphagia, and behavioral changes, and can even lead to Cushing’s syndrome, diabetes mellitus, and immunosuppression after long-term use [34]. In veterinary medicine, phytotherapy is still an emerging field [35], despite the existence of solid evidence regarding the anti-inflammatory benefits of plant extracts such as resveratrol, linoleic fatty acid, and α-linolenic fatty acid [36,37,38].

In this context, delphinidin, an anthocyanidin present in fruits such as maqui, has emerged as a candidate of interest. Maqui, a fruit of the shrub *Aristotelia chilensis*, is endemic to southern Chile and of great importance to indigenous peoples, furthermore, 80% of the anthocyanins it contains are delphinidin derivatives, such as delphinidin-3-glucoside (D3G), which is the natural form in the fruit’s plant matrix [39]. It has been recognized as a “superfruit” because of its exceptional polyphenolic profile, particularly its richness in delphinidins, which confer a superior antioxidant capacity compared to other berries [40,41]. Recent reviews have highlighted that standardized maqui extracts, rich in delphinidin, possess pleiotropic effects, including anti-inflammatory and cardioprotective activities in murine and human models [42,43]. Furthermore, in vitro studies in human and murine cells have suggested that these compounds can modulate inflammation-associated disorders by inhibiting the production of lipid mediators and cytokines [44,45].

While the bioactivity of delphinidin has been extensively characterized in murine and human models, and although there are previous studies on general antioxidant diets in dogs [46], to date, there are no scientific reports evaluating the specific immunomodulatory impact of pure delphinidin derivatives on canine leukocytes. The lack of systematic functional studies characterizing the immunomodulatory effects of purified delphinidin derivatives in canine models represents a critical knowledge gap that limits the design of new therapeutic strategies in veterinary medicine. Therefore, this study aimed to determine the immunomodulatory effect of delphinidin chloride and delphinidin-3-glucoside on the function of canine neutrophils and PBMCs. Generating essential background information to validate its potential use as an adjuvant nutraceutical supplement in the prevention and treatment of inflammatory diseases in this species.

## 2. Materials and Methods

### 2.1. Animals

The study population consisted of 20 canines of both sexes, aged between 2 and 6 years. The inclusion criteria required clinically healthy animals with current prophylactic vaccination and deworming schedules. Health status was validated through a complete clinical examination, hemogram, and biochemical profile. Individuals with evidence of concomitant pathologies or those who had received pharmacological treatments within 30 days prior to the start of the trial were excluded from the study.

Each dog constituted an independent experimental unit (biological replicate), strictly ruling out pooling between individuals. Each functional assay was performed with a biological n of 3 different dogs, due to the number of neutrophils and PBMCs obtained per animal after performing the cell isolation protocol from the extracted blood sample. Ethical animal welfare regulations restrict the maximum safe volume of blood extraction per individual; therefore, it was impossible to perform all the functional assays with a single animal.

All experiments were conducted in accordance with the Guidelines for the Use of Animals in Research of the Universidad Austral de Chile and the National Guidelines on the Use of Experimental Animals of the National Commission for Science and Technology of Chile (Law No. 20.380). Additionally, animal experiments were approved by the Institutional Bioethics Review Committee (Bioethics Report No. 495/2023 and Biosafety Report No. 0020-23).

### 2.2. Experimental Design

The owners of the participating dogs were informed of all procedures and signed an informed consent form after all doubts regarding the study were clarified in a personal interview.

Blood samples were obtained from the patients’ homes to minimize stress using careful handling. Venipuncture was performed on the cephalic vein using a collection set with a 23G x ¾ luer adapter (Greiner Bio-One, Kremsmünster, Austria) and 8.5 mL Vacutainer tubes with ACD anticoagulant (Becton Dickinson, Franklin Lakes, NJ, USA) for cell isolation. Additionally, 1.5 mL of blood was collected using a 3 mL disposable syringe, attached to the collection system with a 23G x ¾ luer adapter and distributed into tubes containing EDTA K3 (0.5 mL), Lithium Heparin (0.5 mL) and coagulation activator (0.5 mL) (Guangzhou Improve Medical Instruments Co, Guangzhou, China) for the performance of a complete blood count and biochemical profile. All samples were processed within no more than 1 h post-extraction. Cellular experiments were performed immediately, and plasma was stored at −80 °C.

### 2.3. Isolation of Blood Cells

Cell isolation was performed using density gradient centrifugation, following the protocol described by Muñoz-Caro et al. (2018) [47], with some modifications. In a 50 mL conical tube, 6 mL of Ficoll-Paque™ PLUS reagent (17144002, Cytiva, Marlborough, MA, USA) was added, and subsequently, 9 mL of canine blood was slowly deposited along the tube walls to avoid mixing phases. Centrifugation was performed at 800× *g* for 45 min at room temperature. The PBMC layer was collected, transferred to a 15 mL conical tube, and washed with RPMI 1640 medium (SH30605.01; Cytiva, USA). On the other hand, the fraction from the bottom of the 50 mL tube containing erythrocytes and neutrophils was subjected to osmotic lysis twice with sterile distilled water and centrifuged at 600× *g* for 10 min at room temperature, followed by two washes with RPMI medium. Cell viability was evaluated by Trypan Blue staining (#T8154, Merck, Darmstadt, Germany), and purity was confirmed by Hemacolor^®^ staining (#1.11661, Sigma-Aldrich, St. Louis, MO, USA), using, in both cases, the cells that had values equal to or greater than 95%. Finally, cell counting was performed using a Neubauer chamber.

### 2.4. Delphinidin

For the experimental assays, two high-purity analytical standards were used: delphinidin chloride (DC) (#PHL89625, Sigma-Aldrich, Darmstadt, Germany) and Delphinidin-3-glucoside (D3G) (#PHL89627, Sigma-Aldrich, Germany). DC y D3G are the two main chemical forms of this flavonoid present in maqui [48], a fruit endemic to southern Chile, of great ancestral importance to native peoples [49]. Chemically, DC corresponds to the anthocyanidin core stabilized as a chloride salt (aglycone), whereas D3G represents an anthocyanin composed of a glucose unit linked by a glycosidic bond to position 3 of the C ring (benzopyrylium) [40,50]. The concentrations used were 50 µM, 100 µM, and 150 µM, based on protocols previously described in studies on other cell types [51,52] as there is no prior data on plasma delphinidin levels in dogs. DC and D3G were handled in a biological safety cabinet, protected from light and were resuspended in dimethyl sulfoxide (DMSO) (#D4540, Sigma-Aldrich, Germany), to a final concentration of 60 mM for DC and 40 mM for D3G. Immediately afterwards, they were aliquoted into amber microcentrifuge tubes, and oxygen was displaced. They were then stored at −80 °C until use. A fresh aliquot was used for each assay without thawing cycles. The working solution was prepared in RPMI 1640 culture medium, resulting in a final trace dilution of DMSO of <0.008% in each experiment, reported below the ranges of toxicity [53,54,55].

### 2.5. MTT Cell Viability Assay

Cell viability was determined by metabolic reduction of 3-(4,5-dimethylthiazol-2-yl)-2,5-diphenyltetrazol (MTT) bromide (# 5224, Biotechne Tocris, Bristo, UK) according to the manufacturer’s instructions. Briefly, 5 × 10^5^ neutrophils were treated with lipopolysaccharide (LPS) 500 ng/mL (# L4391, Sigma-Aldrich, St. Louis, MO, USA), DC 50 µM, DC 100 µM, DC 150 µM, D3G 50 µM, D3G 100 µM, and D3G 150 µM for 15 min, 30 min, and 60 min (Table 1). On the other hand, 5 × 10^5^ PBMC were treated with LPS 500 ng/mL, LPS 1000 ng/mL, DC 100 µM, DC 100 µM + LPS, D3G 100 µM, and D3G 100 µM + LPS for 1, 3, and 6 h (Table 2). The incubation times for neutrophils and PBMCs were determined according to the main time ranges used in the subsequent functional assays. The experiment was performed in a 96-well plate incubated at 37 °C and 5% CO_2_. After the incubation period, the medium was replaced with 100 µL of fresh RPMI and 10 µL of MTT (0.5 mg/mL), and the plates were incubated again for 4 h in the dark. Subsequently, the formazan crystals were solubilized with 100 µL of 0.01N SDS-HCl. Finally, the results were obtained by reading the absorbance at 570 nm using a Varioskan Flash reader (Thermo Fisher Scientific, Waltham, MA, USA).

### 2.6. Determination of MMP-9 Activity

MMP-9 activity was assessed after 5 and 15 min of incubation because the literature describes similar release times for the degranulation of human neutrophils treated with other flavonoids [56,57]. These tertiary granules undergo immediate mobilization via exocytosis, representing one of the first effector responses of neutrophils by degrading the extracellular matrix, thus facilitating extravasation and infiltration into inflammatory sites [15]. For this assay, 5 × 10^5^ neutrophils were stimulated with DC or D3G at 50 µM, 100 µM, and 150 µM, in the absence and presence of the inflammatory stimuli LPS (500 ng/mL) and Platelet-Activating Factor (PAF, #511075, Calbiochem, Darmstadt, Germany) (100 nM). The resulting supernatant was stored at −80 °C until zymography was performed [58]. Subsequently, zymography was performed by loading 10 uL of supernatant on a 10% polyacrylamide gel with 0.2% gelatin, then electrophoresis was performed at 200 V for 1 h. The gels were washed and digested overnight at 37 °C with constant stirring. Finally, the gels were stained with Coomassie Blue and scanned for densitometric analysis using the ImageJ 1.54g software. Densitometry was performed on digital images in TIFF format (8 bits). The colors were then inverted, and rectangular regions of interest (ROIs) of equal dimensions were selected for each lane, generating optical density profiles to quantify the pixel intensity of the bands where MMP-9 exhibited activity. Finally, the area under the curve of the intensity peaks was calculated for each region of interest, corresponding to the unstained light band in the zymography gel.

### 2.7. Measurement of ROS Production

5 × 10^5^ neutrophils were treated with DC and D3G at 50, 100, and 150 µM for 15 min, since this was the moment when other flavonoids produced ROS in human neutrophils [57] and is also a time that supports the biological relevance of this function, which is essential for the immediate neutralization of pathogens. Subsequently, luminol at 80 µM was added, and the cells were incubated for 5 min at 37 °C. Then, PAF (100 nM), the ROS-producing stimulus, was applied [59]. The results were obtained by recording chemiluminescence using a Luminoskan Ascent instrument (Thermo Fisher Scientific, Waltham, MA, USA) by recording a baseline measurement (25 readings) followed by 300 post-stimulus readings.

### 2.8. Indirect Measurement of NETs Release

1 × 10^6^ neutrophils were treated with DC or D3G (50, 100, or 150 µM) and stimulated with phorbol 12-myristate acetate (PMA) for 60 min, a time that coincides with the time range described for human neutrophils treated with flavonoids [60] since it achieves optimal chromatin decondensation and release of DNA networks, allowing the sequestration and neutralization of pathogens as a physiological defense mechanism.

For NET digestion, 20 µL of micrococcal nuclease (#0247S, New England Biolab, Ipswich, MA, USA) was added, and the mixture was incubated for 30 min. After centrifugation at 500× *g* for 5 min, the supernatant was collected and placed in a 96-well plate. PicoGreen reagent (#P7581, Thermo Fisher Scientific, Waltham, MA, USA) (1:200) was added, and fluorescence (excitation 484 nm/emission 520 nm) was measured using a Varioskan Flash reader (Thermo Fisher Scientific, MA, USA) [61].

### 2.9. Phagocytosis Assay

The Vybrant™ Phagocytosis Assay kit (#V-6694, Molecular Probes, Eugene, OR, USA) was used. 5 × 10^5^ neutrophils were loaded into a 96-well plate and incubated at 37 °C and 5% CO_2_ for 1 h. The cells were then treated with DC or D3G at 50, 100, and 150 µM for 5, 15, and 30 min, in triplicate, according to the established times to measure this effect in human neutrophils treated with other flavonoids [62] demonstrating the fundamental role of the neutrophil in the early elimination of pathogens and the clearance of cellular debris in the final stages of the inflammatory response. The medium was then removed, fluorescent bioparticles were added, and the plate was incubated for 2 h. To quench extracellular fluorescence, trypan blue was added, and the plate was incubated for 1 min. The fluorescence of the phagocytosed particles was measured at ~480 nm (excitation) and ~520 nm (emission) using a Varioskan Flash reader (Thermo Fisher Scientific, Waltham, MA, USA). The percentage of phagocytosis was calculated by determining the net fluorescence intensity of each experimental condition, by subtracting the nonspecific fluorescence value corresponding to the cell-free condition (fluorescence of experimental condition—fluorescence of cell-free condition). Finally, the net fluorescence of the DC or D3G experimental conditions was normalized with respect to baseline phagocytosis, which was represented by the net fluorescence of the experimental condition of neutrophils containing only RPMI 1640 medium, corresponding to 100% of the phagocytic capacity of the experiment.

### 2.10. Chemotaxis Assay

This assay evaluated the chemoattractant capacity of DC, D3G or PAF and RPMI 1640 medium corresponded to the negative control. 5 × 10^5^ neutrophils were deposited in the upper chamber of 6.5 mm Transwell^®^ inserts (#3415, Corning, Corning, NY, USA) with a 3.0 µm polycarbonate membrane in 24-well plates. Subsequently, in the lower chamber of the insert, in separate wells, the following experimental conditions were added: RPMI 1640 medium (basal negative control), 100 nM PAF (positive control), DC (50, 100, or 150 µM), or D3G (50, 100, or 150 µM), and their response was evaluated at 30, 60, and 120 min of incubation, according to the time ranges used to evaluate the effect of other flavonoids on human neutrophils [57]. After, the inserts were removed and the cells that migrated through the membrane into the lower chamber of the plate were counted using a CRX41^®^ optical microscope (Olympus, Tokyo, Japan). The number of cells per five fields was averaged for each condition.

### 2.11. Quantification of Cytokines by Enzyme-Linked Immunosorbent Assay (ELISA)

The levels of IL-1β, IL-8, IL-6, and TNF-α in neutrophils, as well as IL-17, IL-4, IFN-γ, TNF-α, IL-6, and IL-1β) in PBMC, were determined using the kits detailed in Table 3. The supernatants of neutrophils and PBMC were treated with 100 µM DC or D3G and stimulated with 500 ng/mL LPS, according to the time ranges used in previous studies for neutrophils [63,64,65,66] and for PBMCs [67,68,69]. All experiments were performed according to the manufacturer’s instructions. Briefly, 96-well plates were sensitized with 100 µL capture antibody overnight. After washing three times with 300 µL, they were blocked with 1% Bovine Serum Albumin (BSA) in Phosphate-Buffered Saline (PBS) for 1 h. 100 µL of samples and standards were incubated in triplicate for 2 h, followed by washing and incubation with the detection antibody for 2 h. Streptavidin-Horseradish Peroxidase conjugate was then added, and the mixture was incubated for 20 min, washed, and developed with 3,3′,5,5′-tetrametilbencidina (TMB) substrate (20 min). The reaction was stopped with 50 µL of stop solution, and the absorbance was measured at 450 nm using a Varioskan Flash reader (Thermo Fisher Scientific, Waltham, MA, USA).

### 2.12. Statistical Analysis

The results were presented in bar charts, where each bar represented the mean ± standard error of the mean (SEM) for each experimental condition. The normality of the distribution and the homogeneity of variance were verified using the Shapiro–Wilk and Bartlett tests, respectively. Data were analyzed using a one-way ANOVA for each independent time point. Tukey’s post hoc test was used to determine statistical significance and correct for multiple comparisons between experimental groups. The analysis considered the average of the technical triplicates for each independent biological replicate (n). A *p*-value < 0.05 was considered statistically significant. All statistical analyses and graphs were generated using GraphPad Prism^®^ software (v. 10; GraphPad Software, San Diego, CA, USA).

## 3. Results

### 3.1. Delphinidin Preserved Cell Viability in Both Canine Neutrophils and PBMCs

**Figure 1:**
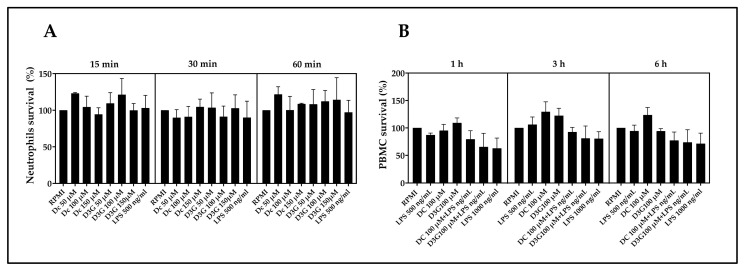
Effect of delphinidin on the viability of canine neutrophils and PBMCs. (**A**) Survival percentage of neutrophils incubated with DC and D3G (50, 100, and 150 µM) or LPS (500 ng/mL) for 15, 30, and 60 min, respectively. (**B**) Survival percentage of PBMCs treated with DC and D3G (100 µM) in the absence or presence of LPS (500 ng/mL and 1000 ng/mL), evaluated at 1, 3, and 6 h. RPMI: 1640 medium. PBMCs: peripheral blood mononuclear cells. DC: delphinidin chloride; D3G: delphinidin-3-glucoside. LPS: lipopolysaccharide. Data are expressed as mean ± SEM of viability percentage relative to control RPMI group (100%), corresponding to three independent assays. Data at each incubation time point were analyzed independently using one-way ANOVA followed by Tukey’s multiple comparisons test.

Cell viability was expressed as the percentage of survival, with the untreated control group RPMI representing 100%. Although the results showed dispersion across all conditions, in the case of neutrophils, where the effects of LPS, DC, and D3G (50, 100, and 150 µM) were evaluated, the survival levels remained at mean values similar to the baseline, showing a homogeneous distribution (Figure 1A). Furthermore, for PBMCs, only the 100 µM concentration of DC or D3G was evaluated, both in the absence and presence of the inflammatory stimulus LPS, as this was the dose used for the inflammatory cytokine assays. The mean survival rates of PBMC for the individual treatments of LPS 500 ng/mL, DC 100 µM, and D3G 100 µM remained close to or slightly above baseline levels at all three time points. However, the DC + LPS, D3G + LPS, and LPS 1000 ng/mL groups of PBMCs at all time points evaluated exhibited numerical values lower than the control, but no statistically significant differences compared to the RPMI group (Figure 1B).

### 3.2. Delphinidin Attenuated the Oxidative Burst and NETosis, While Increasing Chemotaxis in Canine Neutrophils

**Figure 2:**
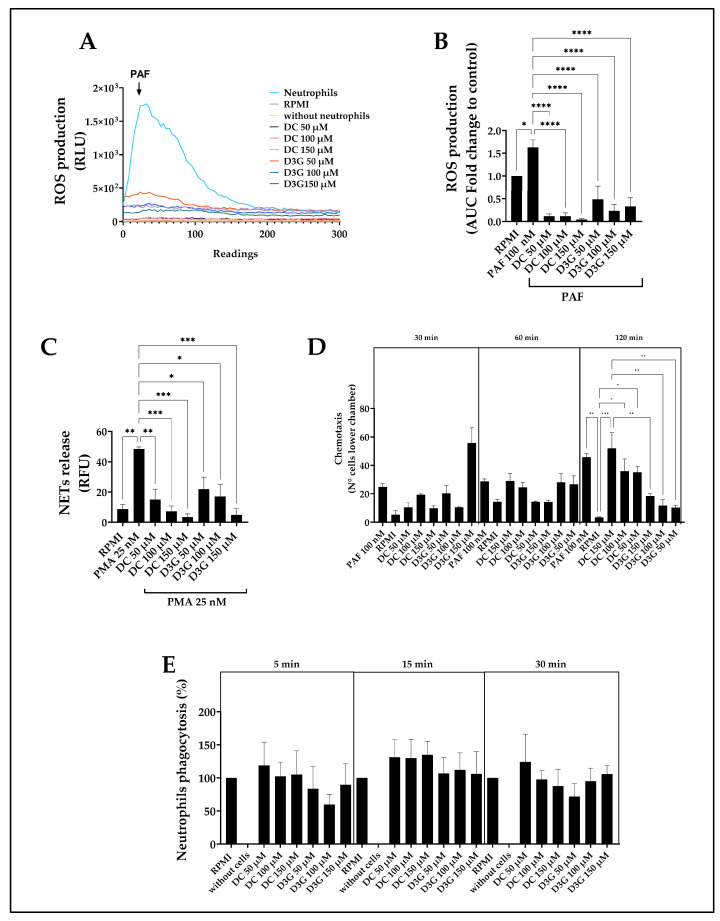
Effect of delphinidin on ROS and NET production, chemotaxis, and phagocytosis in canine neutrophils. (**A**) Representative profiles of ROS generation over 300 s in neutrophils treated with DC or D3G (50, 100, and 150 µM) and stimulated with PAF 100 nM. (**B**) Total ROS production was quantified as the area under the curve (AUC) and expressed as a change relative to the RPMI control. Asterisks indicate significant differences compared to the PAF-stimulated group (**C**) NET release was indirectly quantified by measuring extracellular DNA fluorescence, which is expressed as RFU. Cells were treated with DC or D3G at concentrations of 50, 100, and 150 µM and stimulated with PMA (25 nM). (**D**) Chemotaxis was assessed by quantifying the number of cells that migrated to the lower chamber of a Transwell system after 30, 60, and 120 min of incubation with PAF 100 nM, DC 50 µM, DC 100 µM, and DC 150 µM or D3G 50 µM, D3G 100 µM and D3G 150 µM. (**E**) The percentage of phagocytosis was normalized with respect to baseline phagocytosis (RPMI 1640 medium), set at 100%. Cells were incubated with DC or D3G at 50, 100, and 150 µM for 5, 15, and 30 min, respectively. RPMI: 1640 medium. ROS: reactive oxygen species. DC: delphinidin chloride; D3G: delphinidin-3-glucoside. PAF: platelet-activating factor. RLU: relative luminescence unit. NET: neutrophil extracellular traps. PMA: phorbol 12-myristate 13-acetate. RFU: relative fluorescence unit. Data are presented as mean ± SEM of three independent assays. Data at each incubation time point were analyzed independently using one-way ANOVA followed by Tukey’s multiple comparisons test. * *p* < 0.05, ** *p* < 0.01, *** *p* < 0.001, **** *p* < 0.0001.

ROS production in canine neutrophils was measured in relative luminescence units (RLU) over 300 readings. PAF induced a rapid and robust respiratory burst, reaching a peak intensity above 1.5 × 10^3^ RLU followed by a gradual decline. In contrast, the RPMI and cell-free controls remained near-zero baseline. Similarly, treatment with the compounds DC or D3G at all concentrations tested (50, 100, and 150 µM) when stimulated with PAF showed flat profiles that almost completely overlapped with the baseline control RPMI group. However, 50 µM D3G treatment (orange line) resulted in a slight and transient increase compared to the other treatments (Figure 2A). Figure 2B shows ROS production quantified by the area under the curve (AUC) and normalized as a fold of the control RPMI group, where PAF induced a 1.5-fold increase compared to the baseline control (RPMI, set at 1.0). In contrast, exposure of neutrophils to compounds DC and D3G, at concentrations of 50, 100, and 150 µM, resulted in significantly lower ROS levels compared to the PAF-induced activation of the untreated control RPMI group.

Canine NET formation, quantified by fluorescence, showed that 25 nM PMA induced a significant increase compared to the control RPMI group. However, co-treatment with DC + PMA and D3G + PMA significantly and dose-dependently reduced NET formation compared to treatment with PMA alone. The highest concentration of 150 µM for both compounds showed the greatest inhibitory capacity, which was below the baseline control RPMI group. Similarly, 100 µM and 50 µM of DC and D3G significantly reduced NET production compared to the group treated with PMA alone, although with a slightly lower magnitude of inhibition than the maximum dose (Figure 2C).

The chemotactic capacity of canine neutrophils was evaluated by quantifying the number of cells that migrated to the lower chamber of the transwell insert containing different concentrations of DC, D3G, and 100 nM PAF after 30, 60, and 120 min of incubation. During the 120 min incubation period, a statistically significant increase in neutrophil chemotaxis was observed in response to DC stimulation at all concentrations evaluated (50, 100, and 150 µM) compared to the RPMI group. Furthermore, the DC 150 µM group showed a statistically significant increase in the number of migrating neutrophils compared to the D3G groups at all concentrations (Figure 2D).

The percentage of phagocytosis in canine neutrophils treated with 50, 100, and 150 µM DC and D3G for 5, 15, and 30 min were normalized with respect to basal phagocytosis (RPMI 1640 medium), set at 100%. Although most values in the evaluated groups showed a phagocytosis percentage close to and above 100%, the observed results were not statistically significant (Figure 2E).

### 3.3. D3G Decreases MMP-9 Activity in Canine Neutrophils

**Figure 3:**
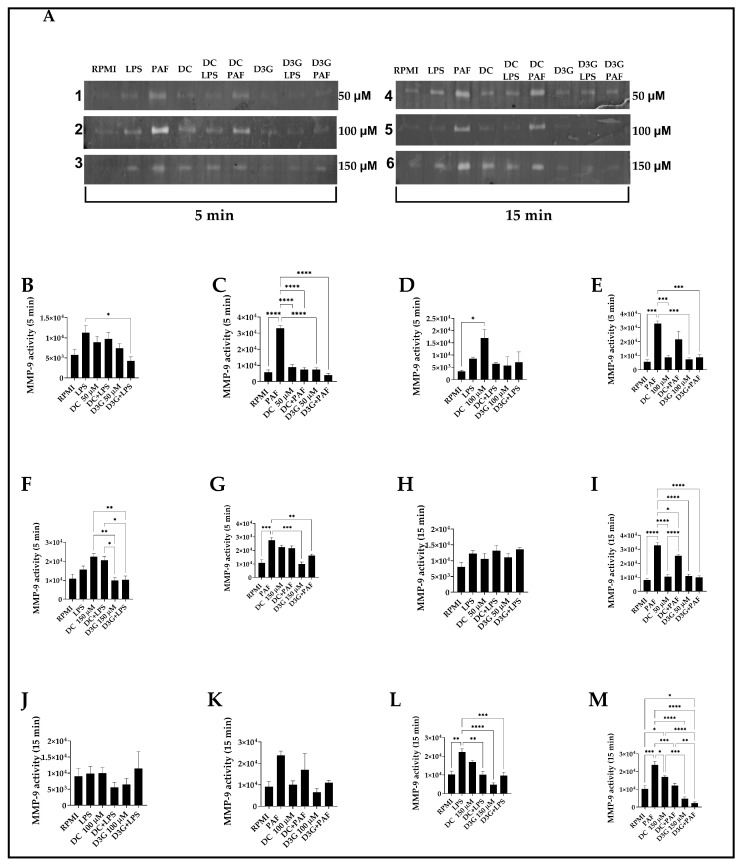
Effect of DC and D3G on MMP-9 activity in canine neutrophils. Neutrophils were incubated for 5 min (**B**–**G**) and 15 min (**H**–**M**) with DC or D3G at concentrations of 50 µM (**B**,**C**,**H**,**I**), 100 µM (**D**,**E**,**J**,**K**), and 150 µM (**F**,**G**,**L**,**M**) in the presence or absence of the inflammatory stimuli LPS (500 ng/mL) (**B**,**D**,**F**,**H**,**J**,**L**) or PAF (100 nM) (**C**,**E**,**G**,**I**,**K**,**M**). The inset in (**A**) shows the zymography gels according to each set of experiments, by incubation time and concentration of DC and D3G. RPMI: 1640 medium. DC: delphinidin chloride, D3G: delphinidin chloride 3-glucoside. LPS: lipopolysaccharide. PAF: platelet-activating factor. Bars represent the mean ± SEM of three independent assays. Statistical analysis was performed using one-way ANOVA followed by Tukey’s multiple comparisons test. * *p* < 0.05, ** *p* < 0.01, *** *p* < 0.001, **** *p* < 0.0001).

Enzymatic activity was detected as the unstained areas of the gel, representing gelatin degradation, visualized as gelatinase activity at 92 kDa, compared to the molecular weight standard. PAF 100 nM significantly increased MMP-9 activity compared to the control RPMI group at all incubation times (5 and 15 min), except for the analysis of the 100 µM concentration of DC and D3G at 15 min of incubation (Figure 3K). When neutrophils were stimulated with PAF 100 µM, D3G significantly decreased MMP-9 activity at all times and concentrations (Figure 3C,E,G,I,M), except for D3G 100 µM + PAF at the 15 min incubation time (Figure 3K). On the other hand, LPS 500 ng/mL only significantly increased MMP-9 activity compared to RPMI 1640 medium, in the 15 min incubation time of the zymography experiment with DC and D3G 150 µM (Figure 3L). Representative zymography gels are shown in insert A of Figure 3, and full gels are available in Supplementary Figure S1.

### 3.4. Delphinidin Modulated Inflammatory Cytokine Production in Canine Neutrophils and PBMCs

**Figure 4:**
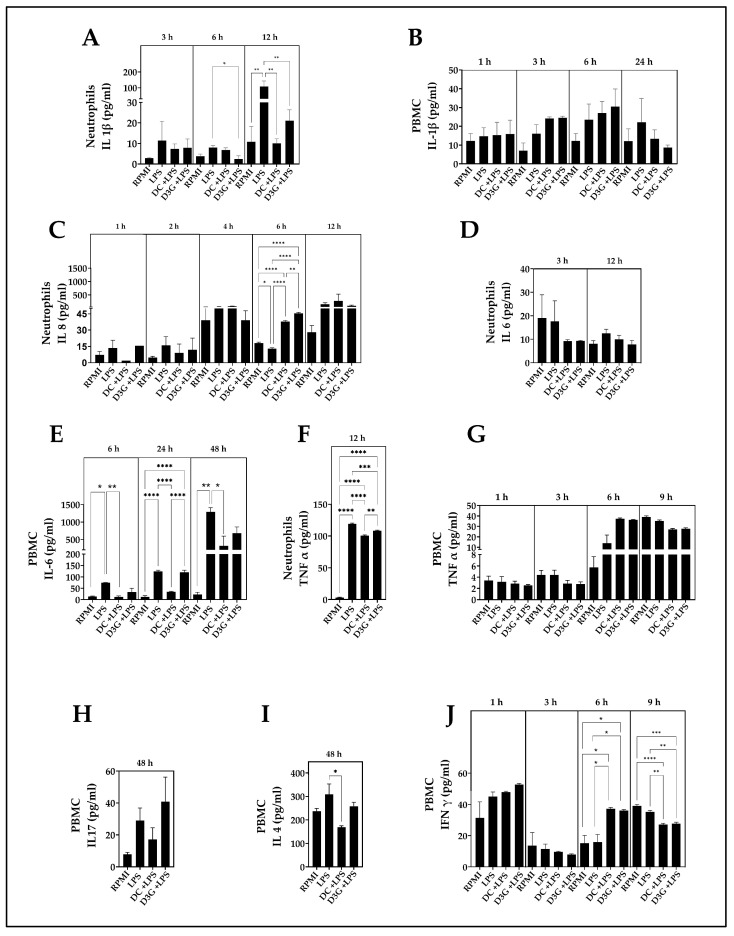
Modulation of inflammatory cytokine secretion by DC and D3G in LPS-stimulated canine neutrophils and PBMCs. Neutrophils and PBMCs were treated with 100 µM DC or 100 µM D3G and subsequently stimulated with 500 ng/mL LPS. Cytokine concentration in the supernatant was quantified by ELISA at different times. (**A**) IL-1β production in neutrophils at 3, 6, and 12 h; (**B**) IL-1β production in PBMCs at 1, 3, 6, and 24 h. (**C**) IL-8 levels at 1, 2, 4, 6, and 12 h. (**D**) IL-6 levels in neutrophils (3 and 12 h). (**E**) IL-6 production in PBMCs (6, 24, and 48 h). (**F**) TNF-α concentration in canine neutrophils at 12 h. (**G**) TNF-α production in PBMC at 1, 3, 6, and 9 h. (**H**) IL-17 levels in PBMCs at 48 h. (**I**) IL-4 levels in PBMCs at 48 h. (**J**) IFN-γ production kinetics in PBMCs at 1, 3, 6, and 9 h. PBMCs: peripheral blood mononuclear cells. RPMI: 1640 medium. DC: delphinidin chloride; D3G: delphinidin-3-glucoside. LPS: lipopolysaccharide. IL-1β: interleukin-1β. IL-8: interleukin-8. IL-6: interleukin-6. TNF-α: tumor necrosis factor-α. IL-17: interleukin-17. IL-4: interleukin-4. IFN-γ: interferon-γ. Data are expressed as mean ± SEM (n = 3). Data at each incubation time point were analyzed independently using one-way ANOVA followed by Tukey’s multiple comparisons test. * *p* < 0.05, ** *p* < 0.01, *** *p* < 0.001, **** *p* < 0.0001).

Quantification of IL-1β levels in the supernatant of canine neutrophils treated with DC + LPS and D3G + LPS revealed different responses to LPS stimulation. At 6 h of incubation, the group treated with LPS + D3G showed a statistically significant lower concentration of IL-1β compared to the LPS group. At 12 h post-stimulation, the group treated with LPS alone exhibited a concentration of approximately 200 pg/mL, representing a significant increase in IL-1β secretion compared to the RPMI. Meanwhile, the DC + LPS and D3G + LPS groups significantly reversed this effect, maintaining cytokine levels within a range comparable to that of the control RPMI group and demonstrating potent inhibitory capacity (Figure 4A). The IL-1β secretion in PBMCs no statistically significant differences were observed (Figure 4B).

At 6 h of neutrophil incubation, IL-8 concentrations in the DC + LPS and D3G + LPS groups showed significantly higher concentration values than in the LPS and RPMI groups (Figure 4C).

The statistical analysis of the effect of delphinidin on IL-6 production of neutrophils did not reveal any significant differences between the groups (Figure 4D). However, IL-6 production in PBMC showed that at 6 h the LPS group had a mean concentration close to 75 pg/mL, which was significantly higher than that of the RPMI group, while the DC + LPS group exhibited significantly lower values than the LPS group. At 24 h, while the LPS group and the D3G + LPS group maintained high levels of cytokines (above 100 pg/mL) with a significant difference compared to the RPMI, the group treated with DC + LPS showed significantly lower concentrations than the LPS stimulation group. The greatest response was observed at 48 h of treatment, at which point the LPS group reached concentrations of 1 × 10^3^ pg/mL, significantly higher than that of the RPMI group, while DC + LPS showed significantly lower IL-6 levels compared to the LPS group, ranging from 300 to 600 pg/mL (Figure 4E).

Quantification of TNF-α 12 h post-stimulation showed that the LPS group reached an approximate mean concentration of 120 pg/mL, which was significantly higher than that observed in the baseline control RPMI group. Compared to the LPS group, the DC + LPS and D3G + LPS groups had significantly lower TNF-α concentrations. Additionally, the DC + LPS group exhibited significantly lower mean levels (close to 100 pg/mL) than the D3G + LPS group; however, despite this reduction observed in the treated groups, both maintained significantly higher concentrations than the control RPMI group (Figure 4F). Regarding TNF-α production in PBMC no statistically significant differences were observed at any of the evaluated time points (Figure 4G).

IL-17 production was assessed in PBMCs supernatants 48 h after treatment; however, statistical analysis did not reveal significant differences between the experimental groups (Figure 4H).

IL-4 levels in PBMCs supernatants at 48 h showed a baseline concentration in the control RPMI group of approximately 240 pg/mL, while the LPS group presented a mean value of approximately 310 pg/mL. When analyzing the effect of the evaluated compounds in response to this stimulus, the group treated with DC + LPS showed a mean concentration lower than 170 pg/mL, which represented a significant decrease compared to the LPS group. Meanwhile, the group treated with D3G + LPS exhibited a mean concentration of approximately 260 pg/mL, with no statistically significant differences observed compared to the group stimulated only with LPS (Figure 4I).

IFN-γ production in PBMCs showed differential behavior at each evaluation period. During the 1 and 3 h treatments, no statistically significant differences were observed between the experimental groups. However, at 6 h, both the DC + LPS and D3G + LPS groups showed IFN-γ levels of approximately 40 pg/mL, significantly higher than those of the LPS and RPMI groups. Finally, at 9 h post-stimulation, both the DC + LPS and D3G + LPS groups showed a significant decrease in IFN-γ concentration compared to the RPMI and LPS groups (Figure 4J).

## 4. Discussion

The present study constitutes the first report demonstrating that purified DC and D3G derivatives exert potent differential immunomodulatory activity in canine neutrophils and PBMCs with a favorable safety profile, characterized by a significant reduction in ROS, NET formation, MMP-9 activity, and the regulation of the production of the proinflammatory cytokines IL-1β, TNF-α, IL-6, and IFN-γ.

First, the evaluation of cell viability using the MTT assay revealed that neither DC nor D3G, at concentrations up to 150 µM, negatively affected neutrophil or PBMC survival. Although PBMCs showed lower values than the control RPMI group when subjected to the inflammatory stimulus of LPS plus DC or D3G, the absence of statistically significant differences suggests that this could represent mild and reversible cellular metabolic stress, within homeostatic limits that do not activate cell death pathways [70,71]. This agrees with reports by Guo et al. (2024) and Ruiz-Cano & Arnao (2024) [37,46], who highlight that plant extracts and polyphenols usually present a high safety margin in canine models, promoting nutritional benefits without associated cytotoxicity. The preservation of viability is an essential prerequisite for any potential immunomodulator, ensuring that the decrease in functional activity, such as ROS production, is due to molecular regulation and not cell death [36].

One of the most critical mechanisms in acute inflammation is the neutrophil respiratory burst. Our results showed that DC and D3G significantly reduced PAF-induced ROS production. This antioxidant effect is consistent with previous studies in dogs, such as that by Woode et al. (2015) [72], who observed that resveratrol (another polyphenol) decreases oxidative burst capacity in canine leukocytes. Furthermore, canine PBMCs treated with purified flavonoids and stimulated with Escherichia coli also showed a decrease in the oxidative response [73]. Likewise, it has been demonstrated in HepG2 cell lines that delphinidin protects against hydrogen peroxide-induced oxidative stress, restoring intracellular redox homeostasis [74]. This free radical “scavenging” ability is an intrinsic property of the chemical structure of Maqui anthocyanins, supporting their potential to mitigate oxidative tissue damage [43]. Furthermore, it has been documented that this potent antioxidant capacity of delphinidin and its glucosides is not limited only to direct radical scavenging but implies functional protection of cell membranes against dyshomeostasis induced by toxic agents, as observed in neuronal models exposed to beta-amyloid peptides [75]. This membrane stabilization mechanism could be key to explaining the preservation of canine neutrophil functionality under oxidative stress conditions observed in our study. Given that excess ROS is fundamental in the pathogenesis of various inflammatory diseases and tissue damage in dogs [14,76], delphinidin’s ability to mitigate this oxidative stress suggests valuable therapeutic potential, similar to what was observed in healthy dogs fed a diet enriched with antioxidants where a significant decrease in plasma levels of reactive oxygen metabolites was evident [77,78] and in dogs with parvovirus enteritis supplemented with antioxidants during their outpatient treatment where oxidative stress was reduced [79].

In addition to the oxidative control RPMI group, a dose-dependent inhibition in PMA-induced NET formation was observed. Initially, this result could be interpreted with caution, as further studies are needed to confirm, through immunofluorescence assays, the colocalization of DNA with specific NET markers, such as myeloperoxidase (MPO) and neutrophil elastase (NE), using microscopy. However, the results of our cell viability assay determined that neither DC nor D3G induced cell death; therefore, the presence of extracellular DNA could be attributed to NET release and not to cell death processes. Although NETs are vital for trapping pathogens, their uncontrolled production is associated with tissue damage and autoimmune diseases [11,18]. The ability of DC and D3G to prevent NETosis positions these compounds as promising agents to limit immunothrombosis and excessive inflammation, an area of growing interest in veterinary medicine [12].

In our evaluation of cell migration, it was determined that DC, at all concentrations, acts as a potent chemoattractant for canine neutrophils, inducing a statistically significant increase in chemotaxis compared to the baseline RPMI control during the 120 min incubation period. This preceded the significant increase in IL-8 release, which occurred 6 h after treatment with DC or D3G. Neutrophils store IL-8 in secretory vesicles that they are able to release into the extracellular environment following early activation, initiating immediate cell migration to continue releasing this cytokine as long as the stimulus is maintained [26,27,80]. Therefore, the early chemotaxis at 2 h, followed by the increase in IL-8 at 6 h, could suggest sophisticated DC-induced immunoregulation, which could eventually offer promising pharmacological and therapeutic prospects compared to traditional anti-inflammatory agents that promote complete immunosuppression. This is because DC and D3G could actively recruit neutrophils while simultaneously inhibiting the oxidative burst, decreasing NETosis, and inhibiting the acute degranulation of tissue-damaging proteases such as MMP-9. To strengthen these results, trials should be incorporated in which neutrophils are pretreated with DC or D3G and exposed to the chemotherapeutic PAF, to further validate our initial results by evaluating the ability of these compounds to modulate neutrophil migration.

Although the use of antioxidant supplements in the diet has been shown to increase phagocytosis in canine neutrophils [81] these effects were not replicated in our study. This difference is likely due to the specific nature of delphinidin or to limitations inherent in experimental design, comparing a nutritional clinical trial with our in vitro laboratory assay.

Regarding tissue remodeling and the activity of MMP-9, an enzyme involved in extracellular matrix degradation and cell migration [15,82], PAF 100 µM was found to be the most potent inducer of MMP-9 production in canine neutrophils, compared to LPS 500 ng/mL. Furthermore, MMP-9 activity was effectively inhibited by D3G following PAF stimulation. However, a pharmacological divergence was observed, as DC at high concentrations (150 µM) significantly increased MMP-9 activity compared to the control RPMI group, while its glycosylated form, D3G, remained below the value of the control RPMI group. This highlights the importance of chemical structure in flavonoid bioactivity and suggests that D3G could offer a more stable therapeutic profile for pathologies where MMP-9 is overexpressed, such as in certain canine cancers or cardiovascular diseases [83]. Unlike previous veterinary research that has used complex extracts of *Aristotelia chilensis*, this work introduces a novel methodological approach by isolating the effect of pure molecules. This has allowed us to elucidate, for the first time using pure molecules in canine neutrophils, that delphinidin glycosylation could have a key functional role in the compound’s stability and safety on critical enzymes such as MMP-9.

Finally, cytokine analysis by ELISA demonstrated that DC and D3G exert pleiotropic anti-inflammatory action. A significant reduction in IL-1β and TNF-α secretion in neutrophils and IL-6, IL-4 and IFN-γ in PBMCs stimulated with LPS was observed. These results agree with investigations describing how delphinidin can inhibit critical inflammatory signaling pathways, such as PI3K/Akt and mTOR, thus reducing proinflammatory cytokine expression in models of psoriatic disease and other chronic pathologies [84]. Delving into the mechanism, studies in T lymphocytes have revealed that delphinidin is capable of activating the nuclear factor of activated T cells (NFAT) through modulation of calcium entry (SOCE), selectively inducing IL-2 production without exacerbating other proinflammatory pathways [52]. This finding is crucial, as it suggests that the immunomodulation observed in canine cells might not be simple global inhibition, but fine regulation of specific intracellular signaling pathways, a mechanism that constitutes an interesting research prospect in future molecular assays of delphinidin with neutrophils and PBMCs. Likewise, aqueous extracts of *Aristotelia chilensis* have demonstrated efficacy in vivo models of atopic dermatitis, suppressing Th2-type cytokines like IL-4 and increasing IFN-γ, which reinforces their potential to restore the altered Th1/Th2 balance in canine cutaneous allergies [85]. Indeed, Maqui extracts have previously demonstrated their ability to inhibit pathogenic interaction between murines macrophages and adipocytes, decreasing the release of inflammatory markers [86]. IL-6 and IL-1β are central mediators in the acute phase response and “inflammaging” in dogs [87,88]. IL-6 inhibition is particularly relevant, given its role in immune dysregulation and septic processes in canines [24,89].

Although the results for TNF-α were variable at short treatment times, the overall pattern of inhibition of Th1 and pro-inflammatory cytokines reinforces delphinidin’s potential to restore immune homeostasis. Similar to observations with other canine dietary immunomodulators: *Withania somnifera* root extract, which produced a significant decrease in serum interferon-γ and TNF-α in geriatric dogs [90]; *Vaccinium myrtillus*, *Curcuma longa*, and *Echinacea angustifolia*, which significantly decreased TNF expression in healthy dogs [91]; a mixture of extracts of *Passiflora incarnata*, *Withania somnifera*, and *Taraxacum officinale* which significantly decreased serum IL6 concentration in senior dogs [92]; and calcifediol, which decreased IL-6 concentrations produced by LPS-stimulated whole blood cultures from healthy dogs [93].

A particularly interesting finding was the biphasic modulation observed in IFN-γ secretion by PBMCs. At 6 h, DC and D3G treatments induced a significant increase in this cytokine, even surpassing the LPS stimulus, to subsequently drastically reduce it below basal levels at 9 h. Although the underlying mechanisms require further characterization, this pattern preliminarily suggests a sophisticated immunomodulatory profile, where it could hypothetically represent an early ‘alert’ Th1-type phase favoring the activation of initial microbicidal mechanisms, followed by a rapid resolution phase preventing chronification of the inflammatory response [94]. This capacity to temporally adjust the response differs from classic anti-inflammatories that usually linearly block signaling and highlights the potential of polyphenols to restore dynamic immune system homeostasis [23,46].

An integral analysis of these findings reveals that modulation exerted by delphinidin transcends simple immunosuppression, suggesting an ‘active resolution’ mechanism of inflammation [11,19]. This modulation could be related to mechanisms observed in metabolic models of obese mice, where Maqui administration not only reduces hepatic and adipose tissue inflammation but improves insulin resistance, suggesting a comprehensive systemic benefit [95,96]. At the molecular level, this metabolic benefit is supported by delphinidin’s ability to inhibit the sodium-glucose cotransporter (SGLT-1) in intestinal epithelium, effectively reducing postprandial glycemia [97]. By controlling hyperglycemia, a key factor of low-grade inflammation is mitigated, indirectly enhancing immune function. This capacity is evidenced in the pharmacological divergence observed on MMP-9, where glycosylation of D3G appears to confer structural stability preventing pro-destructive enzymatic activation observed with the DC at high concentrations; this control is critical, given that MMP-9 dysregulation drives pathological tissue remodeling in canine neoplasms and cardiovascular diseases [82,83]. Furthermore, the proinflammatory cytokine inhibition (12–48 h), particularly the drastic reduction in IL-6 in PBMCs, suggests modulation at the transcriptional level highly relevant for clinical management [89]. By mitigating IL-6, a central biomarker in mortality from sepsis and acute pancreatitis in dogs and controlling ‘inflammaging’ mediators like IL-1β, D3G profiles not only as an anti-inflammatory but as a nutraceutical agent capable of restoring immune homeostasis in both acute and chronic canine pathologies [24,87,88]. Moreover, the therapeutic reach of these compounds could extend to the neuroimmune axis, since recent evidence in cerebral oxidative models indicates that Maqui extracts improve memory and exert neuroprotective effects [98], which is extremely relevant for the management of geriatric canine patients, where cognitive decline and ‘inflammaging’ coexist and feed back into each other.

The potential of delphinidin to modulate the immune response could benefit some dog breeds that exhibit specific immune responses and susceptibilities to immune diseases, such as inflammatory bowel disease in German Shepherd dogs, *Leishmania infantum* infection in Boxer dogs, and canine systemic lupus erythematosus in the Nova Scotia Duck Tolling Retriever [4]. However, this same selective inbreeding resulting from the breeding of breeds that determines specific immune responses could, on the other hand, affect the generalizability of the study results, making the results more representative for mixed-breed dogs.

The results of this research also offer an initial perspective for the study of delphinidin in other animal species where there is dysregulation of neutrophil function in response to infectious agents and immune diseases, for example, respiratory complex and leukosis in cattle [99], acute rhabdomyolysis, infarct hemorrhagic purpura and immune-mediated myositis in equine [100] and pemphigus foliaceus and autoimmune hemolytic anemia in cats [101,102,103]. Thus, the study of the immunomodulatory effects of delphinidin could extend beyond its effects in dogs, positioning it as an emerging therapeutic strategy in animal health.

## 5. Conclusions

The present study constitutes the first report on delphinidin immunomodulation in canine neutrophils and PBMCs. Our findings demonstrated that both delphinidin chloride and delphinidin-3-glucoside confirmed their in vitro safety and orchestrated a comprehensive anti-inflammatory reprogramming, revealed by their ability to control tissue damage through the suppression of ROS and NET production and the regulation of inflammatory cytokines such as IL-1β, TNF-α, IL-6, IL-4, and IFN-γ. Simultaneously, DC facilitated neutrophil recruitment, driven by IL-8 secretion. Crucially, a functional divergence of delphinidin was evidenced, where D3G, unlike DC, maintained stable inhibition of MMP-9 even at high concentrations, demonstrating that glycosylation confers key structural stability. Together, these findings position delphinidin, one of the main bioactive compounds of Maqui (*Aristotelia chilensis*), as a promising nutraceutical potential candidate in animal health, substantiating the need for future clinical studies to validate its efficacy in restoring canine immune homeostasis.

## Figures and Tables

**Table 1:** Experimental design of the MTT Cell Viability Assay of canine neutrophils.

Time	Neutrophils Experimental Conditions							
15 min	RPMI 1640 medium	DC 50 µM	DC 100 µM	DC 150 µM	D3G 50 µM	D3G 100 µM	D3G 150 µM	LPS 500 ng/mL
30 min	RPMI 1640 medium	DC 50 µM	DC 100 µM	DC 150 µM	D3G 50 µM	D3G 100 µM	D3G 150 µM	LPS 500 ng/mL
60 min	RPMI 1640 medium	DC 50 µM	DC 100 µM	DC 150 µM	D3G 50 µM	D3G 100 µM	D3G 150 µM	LPS 500 ng/mL

**Table 2:** Experimental design of the MTT Cell Viability Assay of canine PBMC.

Time	PBMC Experimental Conditions					
1 h	RPMI 1640 medium	LPS 500 ng/mL	DC 100 µM	DC 100 µM + LPS 500 ng/mL	D3G 100 µM	D3G 100 µM + LPS 500 ng/mL
3 h	RPMI 1640 medium	LPS 500 ng/mL	DC 100 µM	DC 100 µM + LPS 500 ng/mL	D3G 100 µM	D3G 100 µM + LPS 500 ng/mL
6 h	RPMI 1640 medium	LPS 500 ng/mL	DC 100 µM	DC 100 µM + LPS 500 ng/mL	D3G 100 µM	D3G 100 µM + LPS 500 ng/mL

**Table 3:** ELISA kits were used for cytokine quantification.

Cytokine	Kit/Brand	Product Code
IL-1β	DuoSet^®^ ELISA Canine IL-1β/IL-1F2 R&D Systems	DY3747
IL-8	DuoSet^®^ ELISA Canine IL-8/CXCL8 R&D Systems	DY1608
IL-6	DuoSet^®^ ELISA Canine IL-6 R&D Systems	DY1609
TNF-α	DuoSet^®^ ELISA Canine TNF-α R&D Systems	DY1507
IL-17	DuoSet^®^ ELISA Canine IL-17A R&D Systems	DY5848
IL-4	DuoSet^®^ ELISA Canine IL-4 R&D Systems	DY754
IFN-γ	DuoSet^®^ ELISA Canine IFN-γ R&D Systems	DY781B

## Data Availability

All data generated or analyzed during the study are included in this published article. The datasets used and/or analyzed during the present research project are available from the corresponding author on reasonable request.

## Supplementary Materials

The following supporting information can be downloaded at https://www.mdpi.com/article/10.3390/ani16050746/s1: Figure S1: Original, complete, uncut zymography gels evaluating the effect of different concentrations and times of DC and D3G on matrix metalloproteinase 9 (MMP-9) activity in canine neutrophils.

## Abbreviations

The following abbreviations are used in this manuscript:

PBMCs	Peripheral blood mononuclear cells
DC	Delphinidin chloride
D3G	Delphinidin-3-glucoside
MTT	3-(4,5-dimethylthiazol-2-yl)-2,5-diphenyltetrazolium bromide
RPMI	Roswell Park Memorial Institute 1640 medium
LPS	Lipopolysaccharide
PAF	Platelet Activating Factor
PMA	Phorbol 12-myristate 13-acetate
ROS	Reactive oxygen species
NETs	Neutrophil extracellular traps
MMP-9	Matrix metalloproteinase-9
RFU	Relative fluorescence unit
RLU	Relative luminiscence unit
