# Supplementary material for: Delphinidin Exerts Immunomodulatory Effects in Canine Neutrophils and Peripheral Blood Mononuclear Cells by Limiting Tissue Damaging Mechanisms and Regulating Cytokine Responses
Source: Animals (Basel). 2026 Feb 27;16(5):746. doi: 10.3390/ani16050746 (PMC12985018; doi:10.3390/ani16050746)
Supplement: Supplementary file 1 [file animals-16-00746-s001.zip › animals-4147748-supplementary.pdf]

Supplementary Materials

A

1 2 3 4 5 6 7 8 9 10

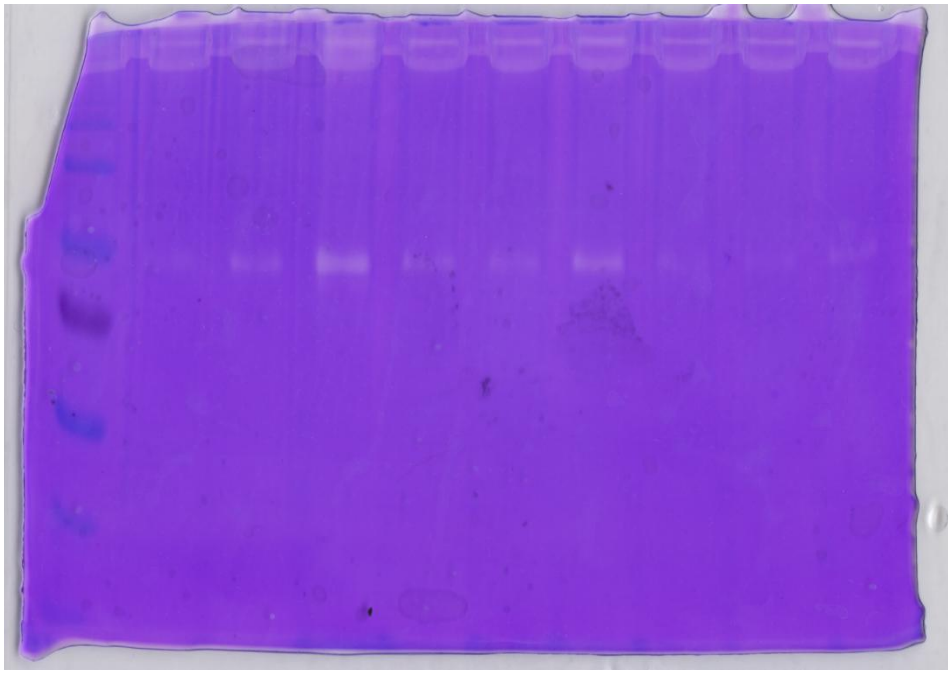

B

1 2 4 5 6 7 8 9 10

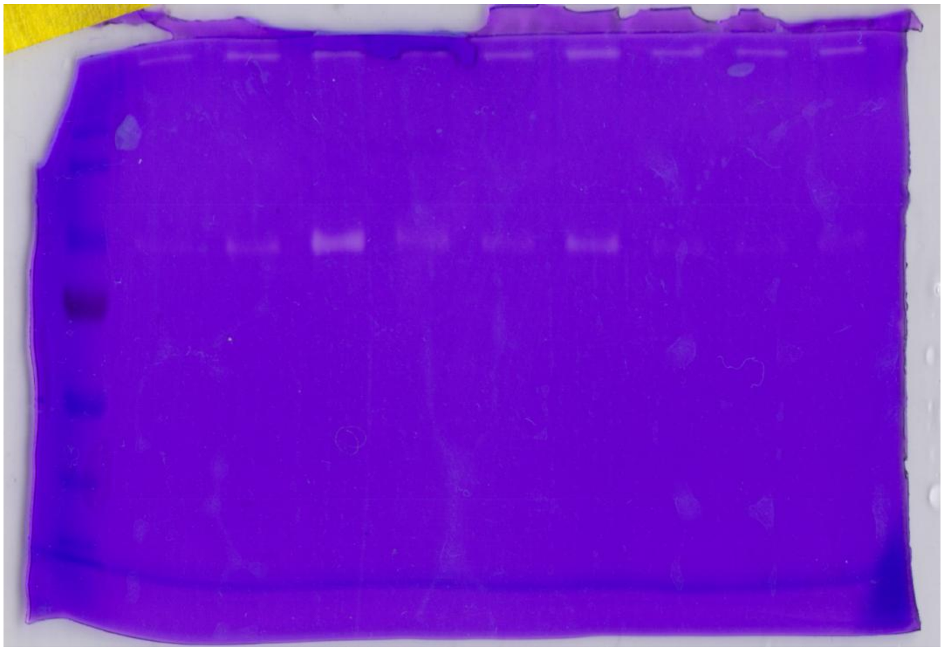

C

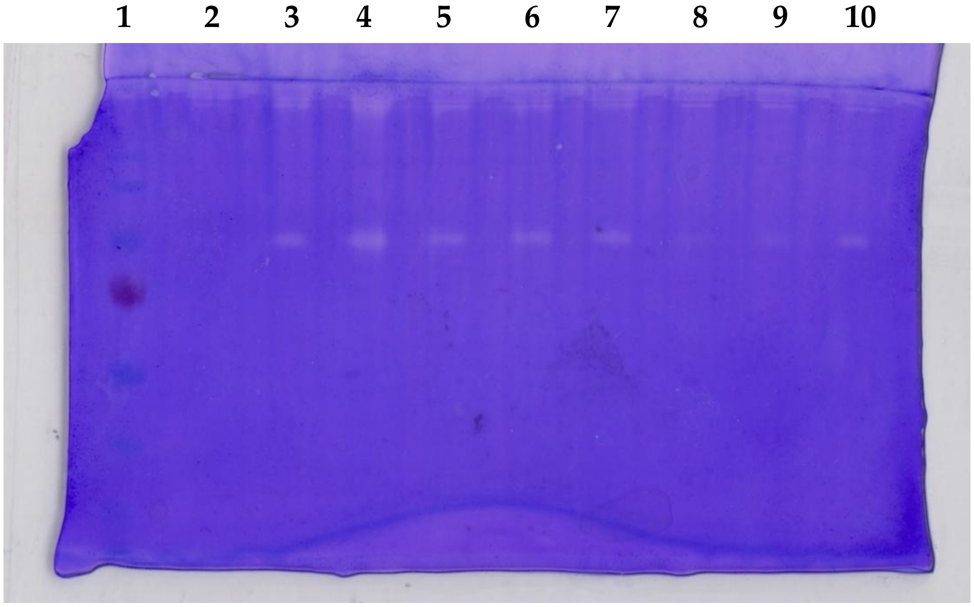

D

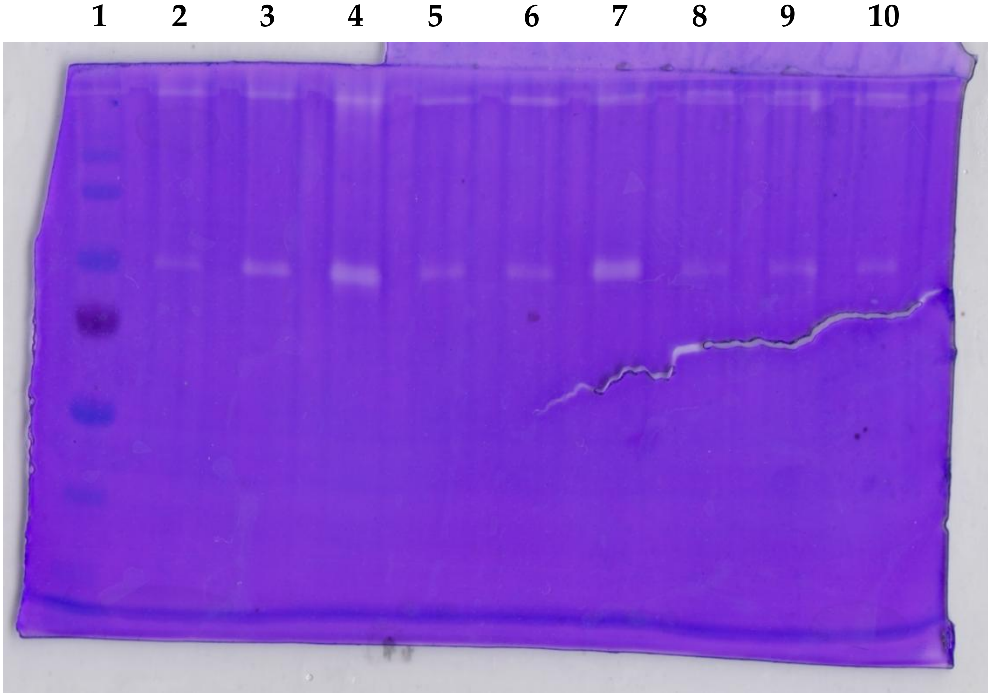

E

1 2 3 4 5 6 7 8 9 10

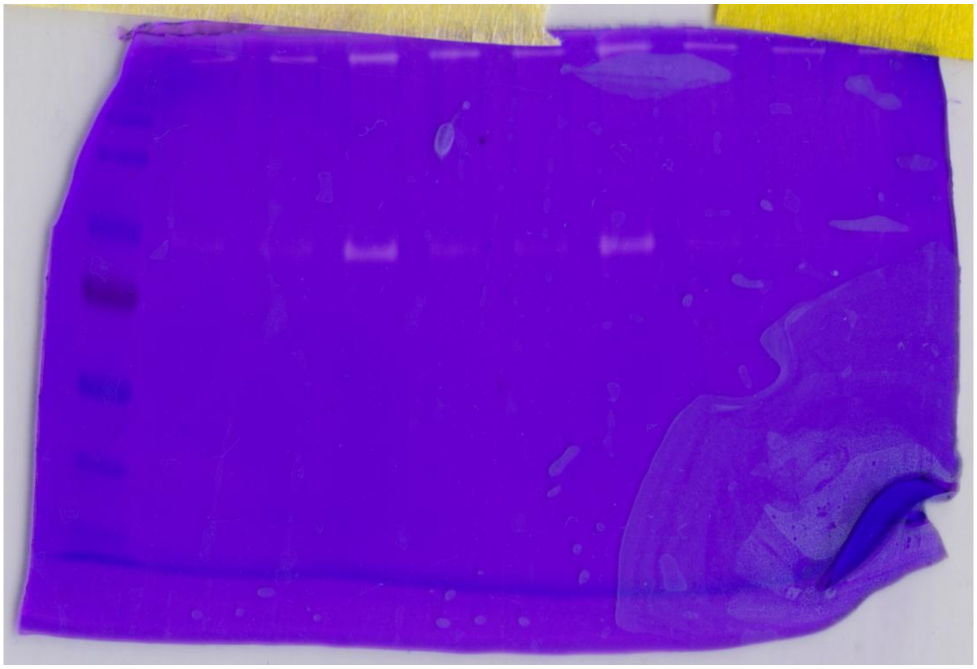

F

1 2 3 4 5 6 7 8 9 10

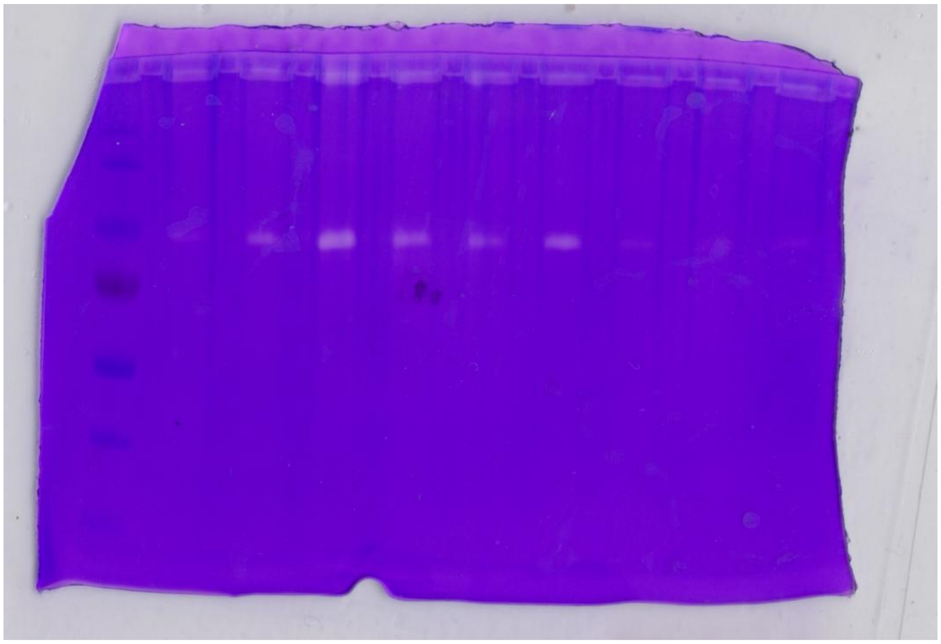

Supplementary figure S1. Original, complete, uncut zymography gels evaluating the effect of different concentrations and times of DC and D3G on matrix metalloproteinase 9 (MMP-9) activity in canine neutrophils. A) Canine neutrophils

stimulated with DC and D3G 50 μM for 5 minutes. 1: pre-stained molecular weight standard. 2: RPMI. 3: LPS 500 ng/ml. 4: PAF 100 nM. 5: DC 50 μM. 6: DC 50 μM + LPS 500 ng/ml. 7: DC 50 μM + PAF 100 nM. 8: D3G 50 μM. 9: D3G 50 μM + LPS 500 ng/ml. 10: D3G 50 μM + PAF 100 nM. B) Canine neutrophils stimulated with DC and D3G 100 μM for 5 minutes. 1: pre-stained molecular weight standard. 2: RPMI. 3: LPS 500 ng/ml. 4: PAF 100 nM. 5: DC 100 μM. 6: DC 100 μM + LPS 500 ng/ml. 7: DC 100 μM + PAF 100 nM. 8: D3G 100 μM. 9: D3G 100 μM + LPS 500 ng/ml. 10: D3G 100 μM + PAF 100 nM. C) Canine neutrophils stimulated with DC and D3G 150 μM for 5 minutes. 1: pre-stained . molecular weight standard. 2: RPMI. 3: LPS 500 ng/ml. 4: PAF 100 nM. 5: DC 150 μM. 6: DC 150 μM + LPS 500 ng/ml. 7: DC 150 μM + PAF 100 nM. 8: D3G 150 μM. 9: D3G 150 μM + LPS 500 ng/ml. 10: D3G 150 μM + PAF 100 nM. D) Canine neutrophils stimulated with DC and D3G 50 μM for 15 minutes. 1: pre-stained molecular weight standard. 2: RPMI. 3: LPS 500 ng/ml. 4: PAF 100 nM. 5: DC 50 μM. 6: DC 50 μM + LPS 500 ng/ml. 7: DC 50 μM + PAF 100 nM. 8: D3G 50 μM. 9: D3G 50 μM + LPS 500 ng/ml. 10: D3G 50 μM + PAF 100 nM. E) Canine neutrophils stimulated with DC and D3G 100 μM for 15 minutes.1: pre-stained molecular weight standard. 2: RPMI. 3: LPS 500 ng/ml. 4: PAF 100 nM. 5: DC 100 μM. 6: DC 100 μM + LPS 500 ng/ml. 7: DC 100 μM + PAF . 100 nM. 8: D3G 100 μM. 9: D3G 100 μM + LPS 500 ng/ml. 10: D3G 100 μM + PAF 100 nM. F) Canine neutrophils stimulated with DC and D3G 150 μM for 15 minutes. 1: pre-stained molecular weight standard. 2: RPMI. 3: LPS 500 ng/ml. 4: PAF 100 nM. 5: DC 150 μM. 6: DC 150 μM + LPS 500 ng/ml. 7: DC 150 μM + PAF 100 nM. 8: D3G . 150 μM. 9: D3G 150 μM + LPS 500 ng/ml. 10: D3G 150 μM + PAF 100 nM.
